# Production of retroviral vectors in continuous high cell density culture

**DOI:** 10.1007/s00253-023-12689-9

**Published:** 2023-08-05

**Authors:** Marc D. Hein, Daniel Kazenmaier, Yasemin van Heuvel, Tanya Dogra, Maurizio Cattaneo, Sascha Y. Kupke, Jörn Stitz, Yvonne Genzel, Udo Reichl

**Affiliations:** 1 Chair of Bioprocess Engineering, Otto-Von-Guericke-University Magdeburg, Magdeburg, Germany; 2 Bioprocess Engineering, Max Planck Institute for Dynamics of Complex Technical Systems, Magdeburg, Germany; 3 Faculty of Biotechnology, University of Applied Sciences Mannheim, Mannheim, Germany; 4 Faculty of Applied Natural Sciences, University of Applied Sciences Cologne, Leverkusen, Germany; 5 Institute of Technical Chemistry, Leibniz University Hannover, Hannover, Germany; 6 Artemis Biosystems, Cambridge, MA USA

**Keywords:** Process intensification, High cell density cultivation, Perfusion cultivation, Gene therapy, Continuous viral vector harvesting, Murine leukemia viral vectors

## Abstract

**Abstract:**

Retroviral vectors derived from murine leukemia virus (MLV) are used in somatic gene therapy applications e.g. for genetic modification of hematopoietic stem cells. Recently, we reported on the establishment of a suspension viral packaging cell line (VPC) for the production of MLV vectors. Human embryonic kidney 293-F (HEK293-F) cells were genetically modified for this purpose using transposon vector technology. Here, we demonstrate the establishment of a continuous high cell density (HCD) process using this cell line. First, we compared different media regarding the maximum achievable viable cell concentration (VCC) in small scale. Next, we transferred this process to a stirred tank bioreactor before we applied intensification strategies. Specifically, we established a perfusion process using an alternating tangential flow filtration system. Here, VCCs up to 27.4E + 06 cells/mL and MLV vector titers up to 8.6E + 06 transducing units/mL were achieved. Finally, we established a continuous HCD process using a tubular membrane for cell retention and continuous viral vector harvesting. Here, the space-time yield was 18-fold higher compared to the respective batch cultivations. Overall, our results clearly demonstrate the feasibility of HCD cultivations for high yield production of viral vectors, especially when combined with continuous viral vector harvesting.

**Key points:**

• *A continuous high cell density process for MLV vector production was established*

• *The tubular cell retention membrane allowed for continuous vector harvesting*

• *The established process had a 18-fold higher space time yield compared to a batch*

**Supplementary Information:**

The online version contains supplementary material available at 10.1007/s00253-023-12689-9.

## Introduction

Retroviral vectors are frequently used for stable gene transduction in somatic gene therapy. The most commonly used viral vectors are derived either from the lentivirus (LV) human immunodeficiency virus type 1 or the gammaretrovirus murine leukemia virus (MLV). In contrast to MLV-derived vectors, LV vectors are capable to transduce a transgene in the genome of non-dividing cells and are therefore preferred for some applications (Cooray et al. 2012; Shin et al. 2015). However, MLV vectors also allow the efficient and stable gene transfer into a wide range of cell types (Li et al. 2018; Maetzig et al. 2011). Currently, the most notable application of MLV vectors is the genetic modification of hematopoietic stem cells (Lundstrom 2018).

Until today, the most common production system for MLV vectors involves the transient triple co-transfection of human embryonic kidney 293-F (HEK293-F) cells with the required envelope, packaging, and transfer vector (Soneoka et al. 1995). As this approach is very time- and cost-intensive, the establishment of a stable MLV vector packaging cell line is advantageous. Until recently, only adherent viral packaging cell lines allowed the production of viral vector titers higher than 1.0E + 06 transducing units/mL (TU/mL) (Rodrigues et al. 2011). Since suspension cell lines generally facilitate scale-up and the implementation of process intensification strategies, they are often preferred for large scale production. However, the use of established suspension packaging cell lines for MLV production always resulted in unsatisfactory viral vector yields, usually below 1E + 06 TU/mL (Chan et al. 2001). To overcome this limitation, the viral vector producing suspension cell line VPC-MSCV-EGFP was generated recently by van Heuvel et al. (van Heuvel et al. 2021). This stable HEK293-F cell-derived VPC was previously shown to yield viral vector titers up to 5.2E + 06 TU/mL in small scale.

Limitations in production capacity of LV vectors, adeno associated viral vectors, and retroviral vectors have been frequently observed. Specifically, the required doses are usually 1E + 11 – 1E + 12 TU/patient (Ansorge et al. 2010; Park et al. 2018), while the average titers in cell-culture based production were in the order of 1E + 6 TU/mL (Ansorge et al. 2009; Sanber et al. 2015; Tomas et al. 2018). Accordingly, significant improvements in viral vector production are required for gene therapy to meet market demands. In the past, various approaches for the intensification of virus particle production processes were described (Gallo-Ramirez et al. 2015; Tapia et al. 2016). One of the most promising options seems to be the implementation of high cell density (HCD) cultures employing perfusion strategies (Hein et al. 2021a; Nikolay et al. 2018; Schwarz et al. 2020; Wu et al. 2021). In a perfusion cultivation, substrate limitations and the accumulation of waste products that are usually observed for conventional batch cultivations can be avoided. Here, using a cell retention system that allows continuous feeding of fresh medium and removal of used medium, much higher viable cell concentrations (VCC) and product titers can be reached. HCD cultures are already used widely for the production of complex or labile proteins, such as enzymes, blood factors, and sometimes monoclonal antibodies (Chotteau 2015; Konstantinov and Cooney 2015). However, for those applications the cultivation is usually carried out as a continuous process, where the product is continuously harvested from the bioreactor through the cell retention device. This offers economic advantages and avoids the risk of process losses by product degradation (Pollock et al. 2013; Tran and Kamen 2022). In the vast majority of those productions, membrane-based cell retention devices were used (Konstantinov and Cooney 2015). For the production of virus particles however, the used hollow fiber membranes (HFMs) usually do not allow for continuous harvesting due to particle size restrictions and membrane clogging (Genzel et al. 2014; Hein et al. 2021a; Nikolay et al. 2020). Therefore, the implementation of a continuous HCD production process was so far only possible using alternative cell retention devices (Manceur et al. 2017). However, since membrane-based cell retention offers some advantages, such as ease of scale-up (Chotteau 2015), it would be desirable to identify HFMs, which enable continuous harvesting of virus particles. Very recently, the use of two of such membranes was described (Hein et al. 2021a; Tran and Kamen 2022). One of them, a tubular membrane called virus harvest unit (VHU), allowed the continuous harvesting of influenza A virus particles.

In the present study, we demonstrate how process intensification strategies could be applied to the production of MLV vectors to drastically increase the viral vector concentration. First, we tested different media in small scale to identify the medium best suited for HCD cultivations. Next, we transferred the production process to a laboratory-scale stirred tank bioreactor (STR) and identified the optimal pH value for cell growth and viral vector production. Next, we evaluated process intensification strategies for the established production process. Specifically, we implemented a perfusion process utilizing a membrane-based alternating tangential flow (ATF) filtration system for cell retention. First, we implemented a perfusion cultivation where the rate was adjusted manually every 24–48 h according to a pre-calculated profile. Subsequently, the perfusion rate was controlled based on online cell concentration measurements of a capacitance probe. Finally, we compared a standard hollow fiber membrane with a VHU for continuous MLV vector harvesting.

## Materials and methods

### Cells

For the production of MLV vectors, a suspension vector packaging cell line was generated as described earlier (van Heuvel et al. 2021). Briefly, *Sleeping Beauty-*derived transposon vectors encompassing the three required vector components, namely a transfer vector (SB-MSCV-EGFP), a packaging construct (SB-gpIpW), and an envelope construct (SBeIhW), were utilized to establish the suspension HEK293-F-derived packaging cell line VPC-MSCV-EGFP.

VPC-MSCV-EGFP cells were maintained in non-baffled shake flasks with a working volume of 40 mL (125 mL plain bottom polycarbonate Erlenmeyer flask, Thermo Fisher Scientific, 4112-0125) at 37 °C, 8% CO_2_ and, 125 rpm (Multitron Pro, Infors HT; 50 mm shaking orbit). Cells were passaged every 3.5 days in one of three media, namely FreeStyle™ 293 Expression Medium (FS; Thermo Fisher Scientific, 12338018), Protein Expression Medium (PEM; Thermo Fisher Scientific, 12661013), and Dynamis™ Medium (DYN; Thermo Fisher Scientific, A2617501). VPC-MSCV-EGFP cells were originally grown in FS, but were adapted to PEM and DYN. For this, a mixture of FS and either of the media was used for cultivation. Here the percentage of DYN or PEM was increased by 25% each passage. After 100% of the medium was either PEM or DYN (after 4 passages), cells were passaged one additional time before being used for the first experiments. Additionally, PEM was supplemented with 8 mM glutamine and 4 mM pyruvate, DYN was supplemented with 8 mM glutamine. To ensure expression of MLV vectors, an antibiotic selection pressure was applied by adding 10 µg/mL puromycin (Thermo Fisher Scientific, A1113803), 200 µg/mL hygromycin (Thermo Fisher Scientific, 10687010), and 200 µg/mL G418 (Thermo Fisher Scientific, 10131027). Antibiotics were added only for the seed train.

For MLV vector quantification in cell based titration assays, adherent murine fibroblast NIH/3T3 cells (ATCC, CRL-1658) and green monkey fibroblast like COS-7 cells (ATCC, CRL-1651) genetically modified to express the ecotropic receptor mCAT (COS-7mCAT) (Berg et al. 2019) were utilized. NIH/3T3 and COS-7mCAT cells were cultivated in high glucose (4.5 g/L) Dulbecco’s modified Eagle’s medium (DMEM) supplemented with 2 mM glutamine and 10% FBS (Gibco, Germany) at 37 °C and 5% CO_2_. Cells were passaged twice a week in tissue culture flasks (175 cm^2^, C7481, Greiner BioOne).

### Cultivation of VPC-MSCV-EGFP cells in shake flasks

To identify the cell culture medium resulting in highest viral vector yields and VCC, screening experiments were conducted in small scale. Prior to inoculation, VPC-MSCV-EGFP cells grown in the three different media (as described before) were centrifuged (300 × g, 5 min, room temperature) and the complete medium was replaced with the corresponding fresh antibiotic-free medium. The VCC was adjusted to 1.0E + 06 cells/mL, before the cell culture broth was transferred to shake flasks with a working volume of 80 mL (250 mL plain bottom polycarbonate Erlenmeyer flask, Thermo Fisher Scientific, 4112–0250). Cells were incubated at 37 °C, 8% CO_2_ and, 125 rpm (Multitron Pro, Infors HT; 50 mm shaking orbit) and were sampled every 12 or 24 h. Part of the sample was used for VCC measurements (Vi-CELL XR, Beckman Coulter, 731050), the rest was centrifuged (3000 × g, 10 min, 4 °C) and the supernatant was aliquoted and stored at -80 °C for real-time reverse transcription qPCRs (real-time RT-qPCR), MLV vector titrations or metabolite analysis (Bioprofile 100 plus analyzer, Nova Biomedical).

### Cultivation of VPC-MSCV-EGFP cells in stirred tank bioreactors

To investigate the applicability of process intensification strategies for MLV vector production, cultivations in STRs (DASGIP® Parallel Bioreactor System, Eppendorf AG, 76DG04CCBB) were performed. The STRs were equipped with one inclined blade impeller (three blades, 30° angle, 50 mm diameter) and a macro-sparger. Prior inoculation, VPC-MSCV-EGFP cells grown in DYN in shake flasks (as described before) were centrifuged (300 × g, 5 min, room temperature) and the complete medium was replaced with fresh antibiotic-free DYN. The STR was inoculated with 0.5E + 06 cells/mL and operated at 37 °C, pO_2_ ≥ 40%, and 250 rpm. The optimal pH value was determined in a screening experiment (pH 6.8–7.4). STRs operated in batch mode had a working volume of 500 mL, perfusion and continuous cultivations were operated with a working volume of 700 mL.

#### Cell retention in perfusion cultivations

For cell retention in perfusion and continuous HCD cultivations, an alternating tangential flow filtration system (ATF 2, Repligen) was used. The flow rate of the diaphragm pump was set to 0.8 L/min, other parameters of the ATF 2 controller were kept as given by the supplier. Two cell retention membranes were investigated for their potential to allow continuous harvesting of MLV vectors. One was a polyethersulfone HFM (0.2 µm pore size, 470 cm^2^ surface area, Spectrum Labs), the other one a tubular VHU (~ 10 µm pore size, 140 cm^2^ surface area, Artemis Biosystems).

#### Perfusion rate control

Perfusion rate control was implemented as described previously (Hein et al. 2021a). Briefly, the addition of fresh medium (feed flow) was controlled by a scale below the STR to maintain a constant working volume. The rate of medium removal (permeate flow) was either (i) adjusted manually or (ii) controlled based on online cell measurements. Manual adjustments were applied every 24–48 h according to a perfusion rate profile, which was calculated based on the cell-specific growth rate and metabolite uptake rates observed for the initial batch cultivations. Changes to the profile were made, when deviations to the expected cell growth were observed (Supplementary Fig. S1). For the continuous HCD cultivation, a multi-frequency capacitance probe connected to a controller (ArcView Controller 265, Hamilton) was used. The signal of the probe was forwarded via an analog 4–20 mA output box (Hamilton) to a peristaltic pump (120 U, Watson-Marlow). This set-up allowed the control of the perfusion rate in accordance to the cell concentrations of the STR. For both control regimes, the target cell-specific perfusion rate (CSPR) was 60 pL/cell/d. For the continuous HCD cultivation, the CSPR was increased to 80 pL/cell/d when a decrease in cell growth was observed. Further, to maintain a steady state, a continuous cell bleed was initiated and the perfusion rate was kept constant when the target VCC of 20.0E + 6 cells/mL was reached. The cell bleed was realized by continuous removal of cell culture broth. The flow rate was chosen in accordance to the cell growth rate calculated when the bleed was initiated.

#### Sampling of the STRs

For batch cultivations, samples were exclusively taken from the cultivation vessel. For the perfusion and continuous HCD cultivations, additional samples were taken from the tubing at the outlet of the cell retention membrane (permeate). Sampling the permeate line also allowed to assess if MLV vectors were passing through the membrane. A part of all samples was used for VCC measurements (Vi-CELL XR, Beckman Coulter, 731050). The remaining sample volume was centrifuged (3000 × g, 10 min, 4 °C), the supernatant aliquoted, and stored at -80 °C for real-time RT-qPCRs, MLV vector titrations, and metabolite analysis (Bioprofile 100 plus analyzer, Nova Biomedical).

### Real-time RT-qPCR

For quantification of the mRNA encoding for the gene of interest (here the reporter gene EGFP) a real-time RT-qPCR was utilized. The mRNA in the cell culture supernatant was purified using a NucleoSpin® RNA virus kit (Macherey-Nagel, 740956) according to the manufacturer´s instructions. A two-step, hot start RT-qPCR with sequence-unrelated tagged primers was used to specifically quantify EGFP mRNA copies. Hot start modification and usage of tagged sequences, prevented measurement of host cell DNA (Kawakami et al. 2011; Lanford et al. 1994). In addition, an external calibration curve was generated. For this, a truncated gag-EGFP sequence was PCR amplified from the transfer vector SB-MSCV-EGFP plasmid (primers: T7-gag/EGFP for 5’-TAATACGACTCACTATAGGGGGCCAGACTGTTACCA-3’ and T7-gag/EGFP rev 5’-TTATCCCGGGTTGTGGCA-3’. The PCR amplified sequences were in vitro transcribed for 2 h at 37 °C using a TranscriptAid T7 High Yield Transcription Kit according to the manufacturer’s instructions, except that 300 ng of PCR product was used in the reaction mix (Thermo Fisher Scientific, K0441). The resulting transcribed RNA standards were treated with 10 vol% DNase (30 min, 37 °C) followed by 10 vol% EDTA treatment (15 min, 65 °C). Next, the RNA standards were purified using a RNA isolation kit (Macherey Nagel, 740955).

In the hot start RT-PCR, 1 µL of EGFP mRNA sample, 0.5 µL of dNTPs, 6.5 µL of nuclease-free water, and 0.5 µL MLV EGFP tagged RT primer (rev 5’-GCTAGCTTCAGCTAGGCATCTTATCCCGGGTTGTGGCA-3’) were first incubated at 65 °C for 5 min and then at 55 °C for 5 min. For cDNA synthesis, 2 µL of 5X RT buffer, 1.25 µL of nuclease free water and 0.25 µL of Maxima H minus reverse transcriptase (ThermoFisher Scientific, EP0751) were added and incubated (30 min, 60 °C), before the reaction was terminated (5 min, 85 °C) (Kupke et al. 2019). A 10-fold serial dilution of the generated RNA standards (5.0E-07–5.0E + 00 ng) was treated analogously to the samples. The generated cDNA was diluted to a final volume of 100 µL.

For the qPCR, 4 µL diluted cDNA, 5 µL of 2X QuantiNova SYBR green PCR mix (QIAgen, 208056), and 0.5 µL each of 1 µM primers MLV EFGP qPCR for 5’- GTCTGCCCTGAGCAAGGAC-3’ and EGFP tagged qPCR rev 5’- GCTAGCTTCAGCTAGGCATC-3’ were mixed. For the real time quantification, samples were subjected to initial denaturation (5 min, 95 °C), before 40 amplification cycles (10 s, 95 °C; 20 s, 62 °C) were carried out. Melt curve analysis was between 65–90 °C (Kupke et al. 2019).

For absolute quantification, a regression curve analysis was formulated by plotting CT values of ten-fold diluted RNA standards against log_10_ number of RNA molecules, as described previously (Kralik and Ricchi 2017).

### MLV vector titration

For quantification of MLV vector titers, adherent cells were transduced and analyzed for EGFP expression. More specifically, NIH/3T3 and COS-7mCAT cells were seeded in 48-well dishes at 1.0E + 04 cells/well in 0.5 mL one day prior to transduction. Next, the medium was removed and viral vector containing supernatant samples in different dilutions in aforementioned expression medium with 10% FBS (total volume 250 µL) were added to the cells and incubated for three days. Three days post transduction, cells were detached and analyzed for their percentage of EGFP-positive cells utilizing flow cytometry (S3e™, Bio Rad). Viral vector titers were calculated as described previously (Salmon and Trono 2007) employing supernatant dilutions resulting in gene transfer efficiencies between 0.8–40.0%.

### Calculations

To allow a fair and unbiased comparison of the different production processes, certain characteristic parameters, namely volumetric productivity (P_v_), space-time yield (STY), and cell-specific productivity (P_c_), were calculated. To enable a high comparability with previous reports, the viral vector titers determined using NIH/3T3 cells, were used for the calculations. For batch cultivations and the perfusion cultivation with a commonly used HFM (retaining the produced viral vectors), the cell culture broth in the STR was harvested. Therefore, the virus titers in the STR (C_STR,t_; TU/mL) at sampling time and the working volume of the STR (V_STR_; mL) were used to calculate the total number of produced viral vectors (N_total_; TU) over the process time.

1 $${N}_{total}={C}_{STR,t}\times {V}_{STR}$$

In case of the continuous HCD cultivation using the VHU, it was expected that MLV vectors would be continuously removed from the STR. For continuous production processes, usually only the permeate is harvested (Konstantinov and Cooney 2015). Therefore, the total number of MLV vectors was calculated based on the viral vector titers in the permeate line (C_Perm_; TU/mL) and the volume of the collected permeate (V_Perm_; mL) over the entire process time.

2 $${N}_{total}=\sum_{i=0}^{i=t-1}\frac{{C}_{perm,t+1}+{C}_{perm,t}}{2}\times ({V}_{perm,t+1}{-V}_{perm,t})$$

The P_v_ (TU/mL/d) describes the number of viral vectors produced over the entire process time (N_total_; TU) per total medium used for a cultivation (V_total_; mL) and cultivation time (t_total_; d). For perfusion cultivations, the volume of the permeate, the cell bleed, and the working volume in the STR needed to be considered for the calculation (Göbel et al. 2022a).

3 $${P}_{v} =\frac{{N}_{total}}{{V}_{total}\times {t}_{total}}$$

The STY (TU/mL/d) describes the number of viral vectors produced over the entire process time per working volume and day (Göbel et al. 2022a). This allows, for example, the comparison of different production processes regarding the number of viral vectors produced within the same time for a given reactor system. However, the total volume of consumed medium is not considered.

4 $$STY =\frac{{N}_{total}}{{V}_{STR}\times {t}_{total}}$$

The P_c_ (TU/cell/d) describes the number of viral vectors produced over the entire process time (N_total_; TU) per cell and day. For this, the integral of viable cells (IVC; cell × day) needs to be calculated based on the cell concentration (VCC; cells/ml) over the process time first (Sauer et al. 2000).

$$\begin{array}{c}IVC=\sum\limits_{i=0}^{i=t-1}\frac{{VCC}_{t+1}+{VCC}_{t}}{2}\times {V}_{STR}\times \left(\left(t+1\right)-t\right)\\ {P}_{c}=\frac{{N}_{total}}{IVC}\end{array}$$

## Results

### High viral vector yield for VPC-MSCV-EGFP cells grown in DYN

To implement a production process for MLV vectors using VPC-MSCV-EGFP cells, first a selection of three media was evaluated for their impact on cell growth and MLV yield. For this, VPC-MSCV-EGFP cells were cultivated in shake flasks (working volume 80 mL) in FS, PEM, or DYN.

**Fig. 1:**
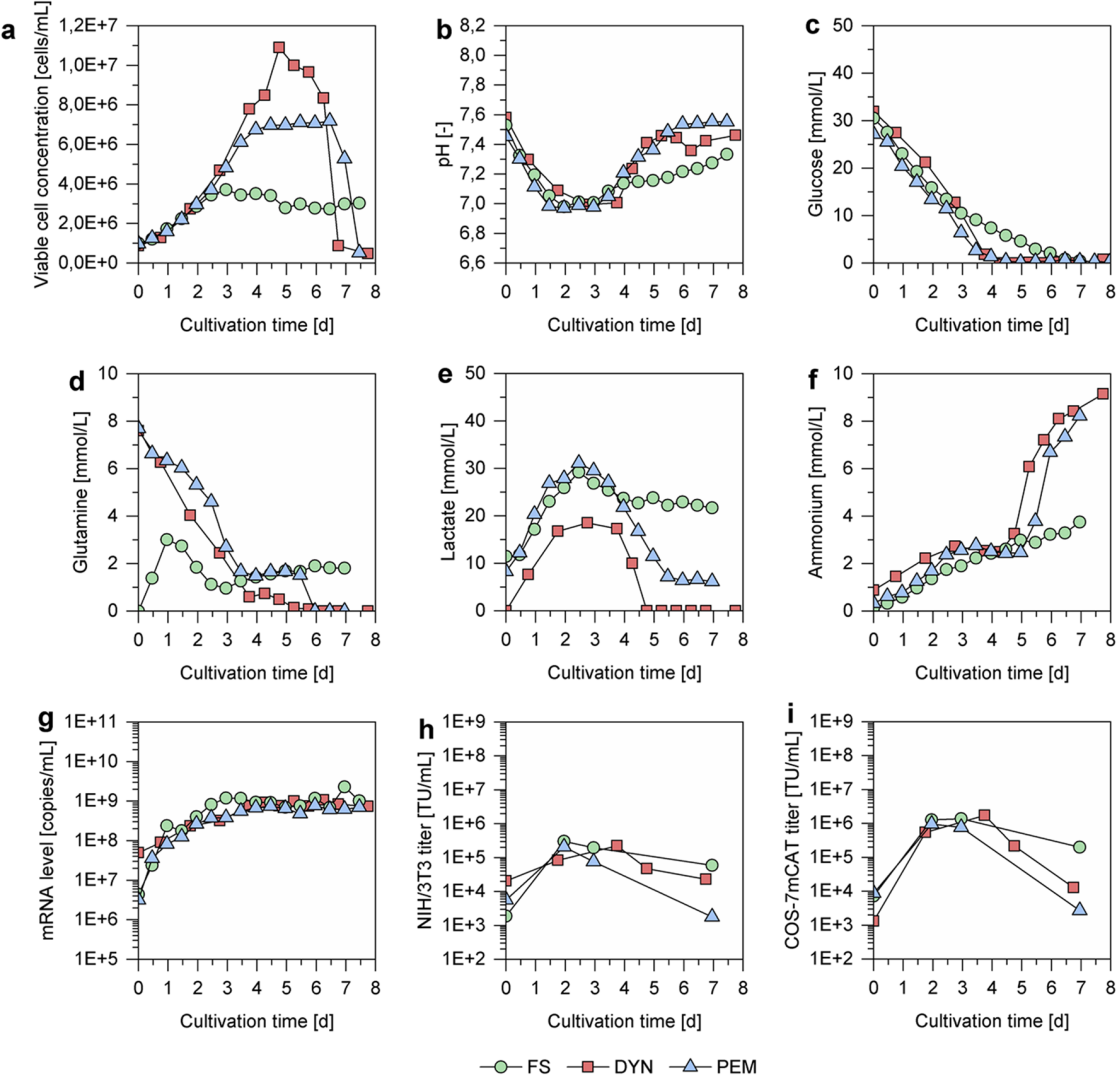
VPC-MSCV-EGFP cell concentrations, pH values, metabolite concentrations, mRNA levels and viral vector titers in small scale culture. Shake flasks (80 mL working volume) were inoculated with VPC-MSCV-EGFP cells at 1.0E + 06 cells/mL in FreeStyle™ 293 Expression Medium (FS; green), Protein Expression Medium (PEM; blue), or Dynamis™ Medium (DYN; red). (**a**) Viable cell concentration, (**b**) pH value (**c**–**f**) metabolite concentrations, (**g**) mRNA levels of the gene of interest, (**h**, **i**) viral vector titers. Viral vector titers were determined by titration in (**h**) NIH/3T3 or (**i**) COS-7mCAT cells

VPC-MSCV-EGFP cells grown in any of the three media showed comparable cell growth for the first 2.5 days (Fig. 1a). The average doubling time in the exponential growth phase was 33.3 h for FS, 30.8 h for PEM, and 27.7 h for DYN. However, the maximum VCC differed greatly. More specifically, cells cultivated in DYN reached a maximum VCC of 10.9E + 06 cells/mL after 4.5 days. This was approximately 1.5-fold and 3-fold higher than the maximum VCC observed for PEM (7.2E + 06 cells/mL) and FS (3.7E + 06 cells/mL), respectively. For all three media, the pH value decreased from 7.6 in the beginning of the cultivation to 7.0 when the maximum VCC was reached, before it increased again (Fig. 1b). The concentrations of glucose and lactate over the time course of the cultivation was similar for all three media with lactate concentrations slightly lower for DYN (Fig. 1c, e). While PEM and DYN were supplemented with 8 mM glutamine, the FS medium contained GlutaMAX, which allowed a gradual release of glutamine. This resulted in strong differences in the observed glutamine concentrations (Fig. 1d). For cultivations with PEM and DYN, a strong ammonium accumulation was observed towards the end of the cultivation when all main substrates were depleted (Fig. 1f). This could not be observed for the cultivation using FS medium.

Despite the differences in cell growth and metabolite concentrations, the maximum mRNA levels and viral vector titers were very comparable for all three tested media (Fig. 1g–i). The maximum mRNA levels for all three cultivation media were around 1.0E + 09 copies/mL. The measured viral vector titers, were approximately 2.0E + 05 TU/mL for transduction of NIH/3T3 cells and 1.0E + 06 TU/mL for COS-7mCAT cells, respectively. As in previous work, not surprisingly, the results for the two cell lines used for quantification differed strongly (van Heuvel et al. 2021). Only selected sample time points (inoculum, exponential growth phase, maximum VCC, cultivation end) were chosen for further analysis.

In summary, strong differences in cell growth were observed for the different media, with cells grown in DYN medium showing the highest VCC and the lowest doubling time. Surprisingly, the differences in VCC did not translate to higher mRNA levels and viral vector titers, as all tested media resulted in comparable titers. However, also the scalability of the process needed to be considered. Specifically, cells grown in FS showed very poor cell growth in the STR in preliminary experiments (data not shown). On the other hand, strong viral vector degradation was observed for PEM medium after the maximum titer was reached. As cells grown in DYN showed not only superior doubling times and maximum VCC but also a relatively low viral vector degradation rate, this medium was chosen for subsequent STR cultivations.

### Improved VPC-MSCV-EGFP cell growth in the STR at pH 7.0

In a next step, the production process was transferred to a STR. To evaluate if cell growth could be further improved, different pH values were tested. For shake flask cultivations the pH value was between 7.0 and 7.6. Since pH values as high as 7.6 can be difficult to maintain for HCD cultivations due to the cellular release of CO_2_, the impact of the pH value was only investigated in the feasible range from 6.8 to 7.4. Other process parameters (e.g. pO_2_ level and agitation speed) were chosen according to HEK293-F cultivations previously performed in our laboratory (Göbel et al. 2022b).

**Fig. 2:**
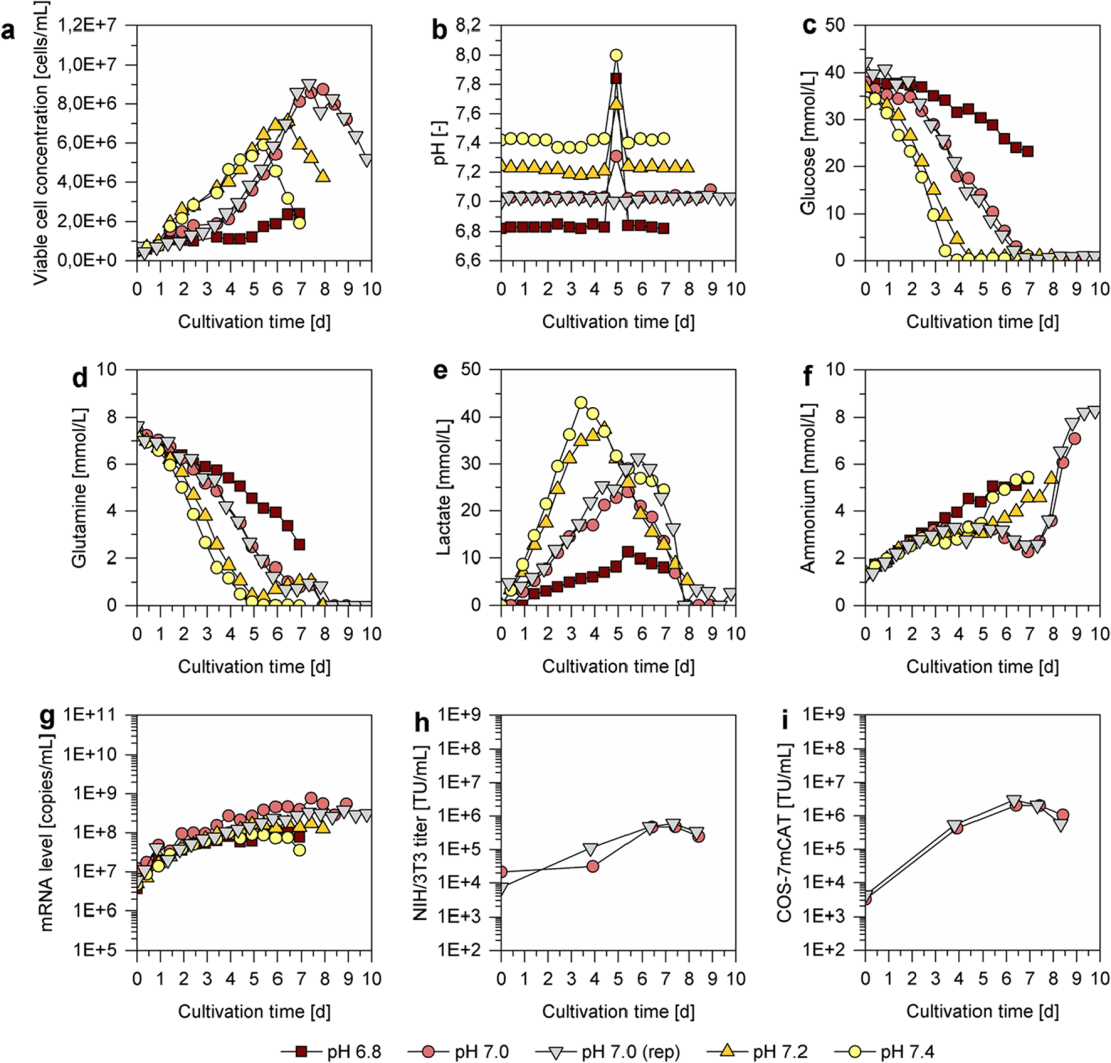
VPC-MSCV-EGFP cell concentrations, pH values, metabolite concentrations, mRNA levels and viral vector titers in 1 L stirred tank bioreactors (STRs) operated at different pH values. STRs (500 mL working volume) were inoculated with VPC-MSCV-EGFP cells at 0.5E + 06 cells/mL in DYN and operated at four different pH values: 6.8 (red), 7.0 (light red and grey), 7.2 (orange), 7.4 (yellow). Four STR cultivations were carried out in parallel, pH 7.0 (rep) was carried out individually as a replicate. At day 5, the pH control of the four parallel-cultivated STRs was interrupted for approximately 6 h due to a malfunction in the CO_2_ supply. (**a**) Viable cell concentrations, (**b**) pH value, (**c**–**f**) metabolite concentrations, (**g**) mRNA levels of the gene of interest, (**h**, **i**) viral vector titers. Viral vector titers were determined by titration in (**h**) NIH/3T3 or (**i**) COS-7mCAT cells only for selected pH7.0 samples

As expected, the cultivation pH had a strong impact on cell growth. Almost no cell growth was detected for pH 6.8 (Fig. 2a) and is therefore not further discussed in the following. For all other pH values, a higher doubling time and a reduced maximum VCC was found compared to shake flask cultivations. More specifically, the average doubling time for cultivations in the STR (pH 7.0–7.4) was between 30 and 35 h in the exponential growth phase and 27.7 h in the shake flask cultivations with DYN. Furthermore, for the cultivation operated at pH 7.0, a lag phase in the beginning of the cultivation, lasting for approximately three days, was observed. This lag phase and the overall cell growth dynamics were reproducible, as shown by a second cultivation in the STR at pH 7.0 (pH 7.0 (rep); Fig. 2a). Surprisingly, the lag phase in the beginning of the cultivation was not observed for cells cultivated at pH 7.2 or 7.4. Nevertheless, the highest maximum VCC was observed at pH 7.0 with 8.8E + 06 cells/mL, which was almost 20% higher than the maximum VCC for the cultivation performed at pH 7.2. The lower maximum VCC for cultivations at pH 7.2 and 7.4 was accompanied with differences in the metabolic profile. Here, a higher glucose uptake (Fig. 2c), glutamine uptake (Fig. 2d), and lactate formation (Fig. 2e) compared to the cultivation at pH 7.0 was observed. As for the shake flask cultivation, high ammonium concentrations were observed towards the end of cultivation for all pH values (Fig. 2f).

After a cultivation time of 5 days, a malfunction in the CO_2_ supply resulted in a shift in the pH values, which lasted for approximately 6 h (Fig. 2b). However, this shift did not significantly influence cell growth, as shown by the high similarity between the two STR cultivations operated at pH 7.0, where for the second cultivation no disturbances in the pH control occurred (Fig. 2a).

Cultivations resulting in higher maximum VCCs also resulted in higher maximum mRNA levels (Fig. 2g). Consequentially, cultivations operated at pH 7.0 yielded the highest mRNA levels, comparable to the results in shake flasks. Since, the viral vector quantification was very labor-intense it was decided to determine it only for the two cultivations operated at pH 7.0 at selected time points (Fig. 2h, i). The maximum viral vector titers, as determined by titration experiments either in NIH/3T3 or COS-7mCAT cells, were very comparable to shake flask cultivations. Overall, the titers in the STR were slightly higher. However, the maximum viral vector titer was only achieved after 7.5 days, 3.5 days later than in shake flask experiments. This was likely caused by the lower inoculum, the initial lag phase, and the higher doubling time observed for cells grown in STR.

When the cultivation pH for subsequent experiments needed to be chosen, pH 7.0 and 7.2 were considered. Cells cultivated at pH 7.2 showed higher cell-specific substrate uptake and waste product formation rates compared to cells cultivated at pH 7.0. More specifically, the glucose uptake rate was 58%, the lactate formation rate was 85%, and the glutamine uptake rate was 47% higher. Consequentially, a higher CSPR of 95 pL/cell/d (compared to 60 pL/cell/d for pH 7.0) would have been required to conduct a perfusion cultivation at pH 7.2. Further, the doubling time during the exponential growth phase (after the initial lag phase) was lower for cells cultivated at pH 7.0 (31.9 h) compared to cells cultivated at pH 7.2 (35.4 h). Lastly, the mRNA levels were higher for cultivations operated at pH 7.0. Therefore, a cultivation pH of 7.0 was chosen for subsequent experiments to explore the potential of process intensification strategies.

### Perfusion HCD cultivations resulted in high viral vector titers

Using the previously determined process parameters, cultivation in perfusion mode at HCD was tested. In a first step, a perfusion cultivation with a manually adjusted perfusion rate and a commonly used HFM for cell retention was implemented (in the following named “perfusion cultivation”). For this cultivation, the perfusion rate was adjusted every 24–48 h according to a pre-calculated profile. The profile was based on the cell-specific growth rate and the glucose uptake rate of previously performed batch cultivations at pH 7.0 and was adapted to the cell growth observed during the perfusion process (Supplementary Fig. S1). In a second step, a control of the perfusion rate based on monitoring the cell concentration in the STR was implemented using an online capacitance probe. For this cultivation, a tubular membrane, called VHU, was used for cell retention and investigated for its potential to allow for continuous viral vector harvesting. Further, this cultivation was performed at a constant cell concentration via a cell bleed. With this, a continuous HCD production process was implemented (in the following named “continuous HCD cultivation”).

**Fig. 3:**
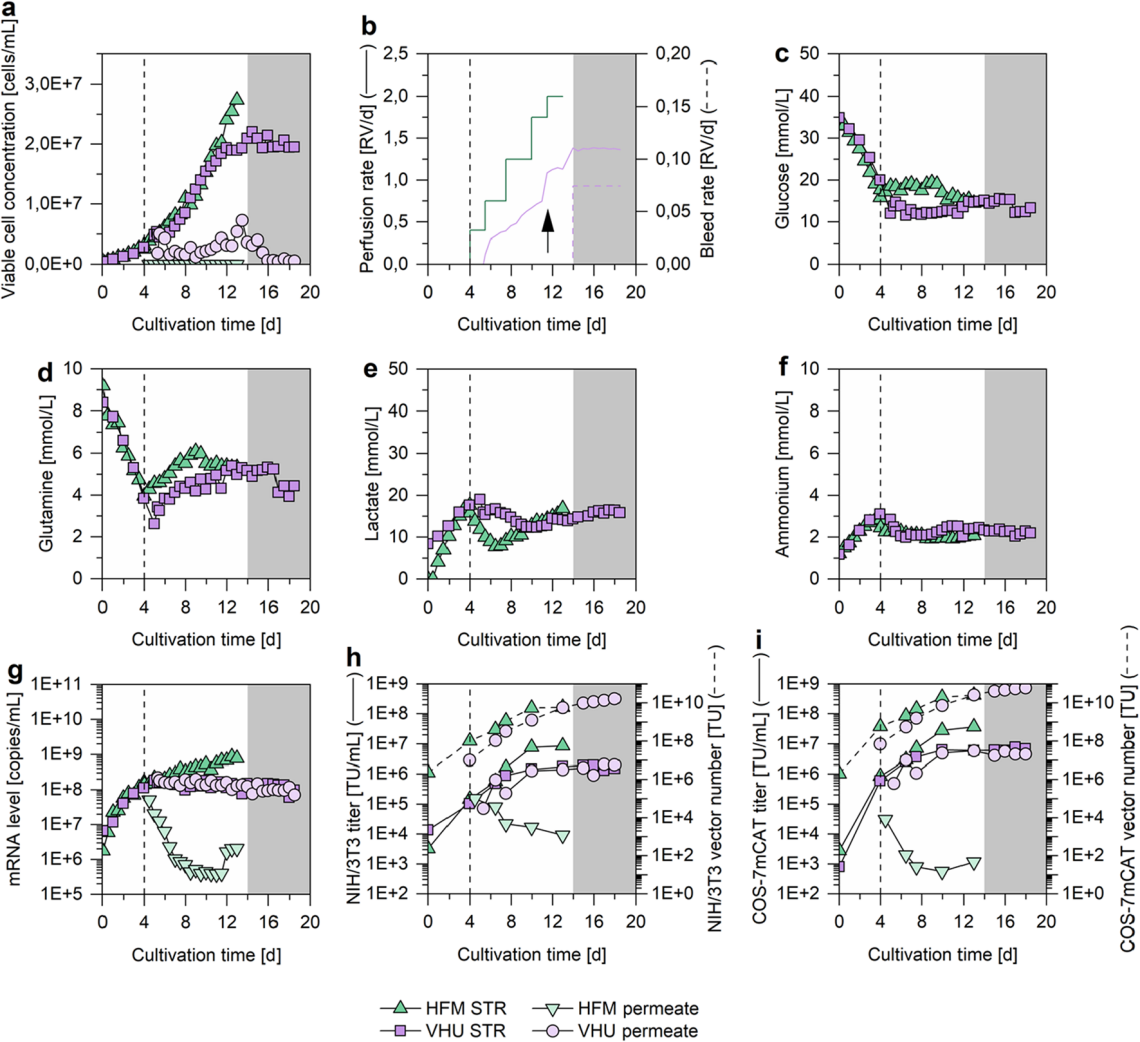
VPC-MSCV-EGFP cell concentrations, metabolite concentrations, mRNA levels and viral vector titers in 1 L stirred tank bioreactors (STRs) coupled to an alternating tangential flow filtration system (ATF). STRs (700 mL working volume) were inoculated with VPC-MSCV-EGFP cells at 0.5E + 06 cells/mL. For cell retention, a hollow fiber membrane (HFM; green, “perfusion cultivation”) or a tubular virus harvest unit (VHU; purple, “continuous HCD cultivation”) was used. The ATF was started after 4 days (HFM; dashed line) or 5 days (VHU; not indicated). The perfusion rate was adjusted manually (HFM) or controlled based on capacitance measurements (VHU). For the VHU, the cell-specific perfusion rate was increased from 60 to 80 pL/cell/day after 11.5 days (black arrow). After 14 days, a continuous cell bleed was started and the perfusion rate was kept constant (grey box). (**a**) Viable cell concentration, (**b**) perfusion and bleed rate, (**c**–**f**) metabolite concentrations, (**g**) mRNA levels of the gene of interest, (**h**, **i**) viral vector titers and vector numbers. Viral vector titers were determined by titration in (**h**) NIH/3T3 or (**i**) COS-7mCAT cells. Light grey and light purple colors indicate mRNA level and viral vector titer in the permeate. Vector numbers are shown with dashed line

Despite the different perfusion rate control regimes and the use of other cell retention membranes, both cultivations showed comparable cell growth (Fig. 3a). However, for both cultivations a reduction in cell growth was observed after the ATF mode was started. More specifically, the doubling time of both cultivations was 36.8 h during the initial batch phase and decreased to an average of 66.5 h after the cultivation was switched to perfusion mode. Nevertheless, both perfusion rate control regimes resulted in sufficient substrate and low waste product concentrations (Fig. 3c–f). This clearly demonstrated the feasibility of the capacity measurement-based perfusion rate control for the given production system. Minor differences in the metabolite concentration could be explained by different durations of the initial batch phase. More specifically, in an attempt to reduce the negative impact of the ATF on cell growth, it was started 24 h later for the cultivation using the VHU (Fig. 3b). However, this did not result in a significantly improved cell growth.

For the perfusion cultivation (HFM used for cell retention), no cells could be observed in the permeate line (Fig. 3a). In contrast, for the continuous HCD cultivation (VHU used for cell retention) relatively high cell concentrations were observed in the permeate line. This is in strong contrast to previous findings of our group (Hein et al. 2021a). Unfortunately, the supplier of the VHU had to change to a new manufacturer of the membranes as a result of the COVID pandemic. The membranes used here were the first membranes from this new supplier. Based on our results the manufacturer has now optimized the manufacturing parameters to be closer to the original membranes used before (17). Measurements from the inner diameter have confirmed this. Therefore, future membranes should not show cells in the permeate if they are larger than 10 µm in diameter. For the described application here however, this was not too much of an issue, as a cell bleed was intended anyway. The cell leakage was very pronounced in the beginning of the cultivation. Here, the VCC in the permeate line approximately equaled the VCC in the STR. However, over the cultivation time, the cell leakage stabilized at a VCC of around 2.0E + 06 cells/mL. When a reduction in the cell growth rate was observed, the CSPR was increased from 60 to 80 pL/cell/day, which resulted in a higher flow through the membrane. Shortly after, an increase in cell leakage was observed again (Fig. 3a, b). This leakage was so strong (VCCs up to 7.3E + 06 cells/mL), that for about 1.5 days barely any increase in the VCC in the STR was observable. After this, the cell leakage reduced and the VCC in the STR started increasing again. These data clearly demonstrate the tremendous impact of a correct membrane design.

For the perfusion cultivation it was expected that the HFM would also retain all produced MLV vectors. Therefore, after the target VCC of almost 30.0E + 06 cells/mL (27.5E + 06 cells/mL) was reached, the process was terminated and the STR was harvested (Fig. 3a). In case of the continuous HCD cultivation with the VHU, it was expected that it would not retain any MLV vectors and that the viral vector concentration in the permeate line should be equivalent to the concentration in the STR. Therefore, after the respective target VCC of 20.0E + 06 cells/mL was reached, a continuous cell bleed was initiated and the perfusion rate was kept constant to maintain a steady state (Fig. 3a, b).

Indeed, the HFM retained most produced MLV vectors and mRNAs. After 10 days (maximum viral vector titer), both titer and mRNA level in the permeate line were three orders of magnitude lower than in the STR (Fig. 3g–i). In strong contrast, when the VHU was used, the viral vector titers and mRNA levels in the permeate line always equaled those in the STR. Consequentially, as viral vectors were continuously removed from the STR, the titers and mRNA levels were lower compared to the perfusion cultivation using the HFM for cell retention (Fig. 3g–i). However, the number of collected viral vectors for the VHU (only the permeate was considered) was ultimately higher than for the HFM (only the STR was considered). Here, longer process times favor the VHU, as the amount of collected viral vectors kept increasing while it stagnated for the HFM after a cultivation time of 10 days (Fig. 3h–i). This clearly demonstrated the feasibility of continuous viral vector harvesting and should allow for the implementation of a fully continuous HCD production process for MLV vectors in future applications.

### Perfusion cultivations have a higher STY than batch cultivations

To assess whether intensified cultivations represent more efficient processes, certain characteristic parameters, namely P_v_, STY, and P_c_, were calculated based on the viral vector titers determined by titration of samples in NIH/3T3 cells (Table 1). Overall, for the performed cultivations, the P_v_ was very comparable. In contrast, the STY was increased 9-fold for the perfusion cultivation using the HFM compared to the average of both batch cultivations. For the continuous HCD cultivation using the VHU, an 18-fold increase was achieved. In other words, the total amount of MLV vectors produced during an entire batch cultivation (assuming an increased working volume of 700 mL) was harvested every 5.7 h for the continuous HCD cultivation with the VHU, once the steady state was reached. This is partially explained by the P_c_, which was almost 5-fold increased for the continuous HCD process compared to the batch processes.

**Table 1:**
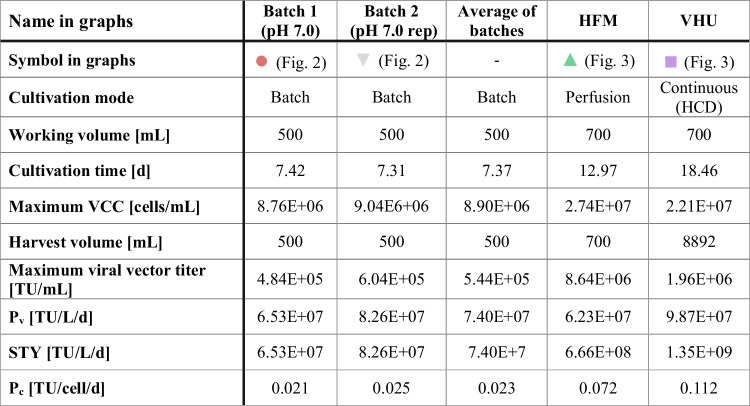
Comparison of different stirred tank bioreactor (STR) cultivations for production of MLV vectors

STRs were operated in batch mode (batch 1 and 2), in perfusion mode using a hollow fiber membrane (HFM), or in fully continuous mode using a tubular virus harvest unit (VHU) for cell retention. The cultivation time is given as time until the maximum titer is reached (batch 1 and 2) or when the process was terminated (HFM and VHU). Either the STR content (batch 1, batch 2 and HFM) or the collected permeate (VHU) was harvested. Maximum viral vector titers, volumetric productivity (P_v_), space-time yields (STY), and cell-specific productivities (P_c_) were calculated based on viral vector titers determined by titration in NIH/3T3 cells

The calculated characteristic parameters clearly demonstrate the advantage of perfusion cultivations and continuous HCD cultivations in particular. Here, more efficient processes with drastically increased product yields could be established by applying intensification strategies.

## Discussion

In this study, we developed a cell culture-based production process for MLV vectors using the HEK293-F cell-derived suspension packaging cell line VPC-MSCV-EGFP. First, the impact of different media on cell growth and maximum viral vector titer was investigated in shake flasks. Next, the process was transferred to a STR and the optimal pH value for cell growth and viral vector yield was determined. Further, a HCD perfusion process using an ATF system for cell retention was established. Lastly, a perfusion rate control using a capacitance probe and continuous viral vector harvesting using a novel VHU were implemented. The developed continuous HCD cultivation with continuous viral vector harvesting was maintained in a steady state for 5 days and yielded a very high number of MLV vectors.

### VPC-MSCV-EGFP cells could be cultivated in HCD perfusion cultivations

We recently reported on the generation of the suspension cell line VPC-MSCV-EGFP, which yielded MLV vector titers up to 5.2E + 06 TU/mL (in NIH/3T3 cells) in small scale (van Heuvel et al. 2021). In shake flask experiments conducted in the present study, vector titers of around 2.0E + 05 TU/mL were observed. The reason for the lower viral vector titers is uncertain, but might be caused by differences in sample preparation (i.e. an additional freeze-thaw step and higher centrifugation force). This could suggest that the viral vector titer reported in the present study underestimates the titer compared to previous literature. However, the viral vector titers within this study can be compared directly, and the maximum vector titer was drastically improved when process intensification strategies were applied. In particular, we showed that VPC-MSCV-EGFP cells cultivated in perfusion cultivations grew to a VCC of up to 27.4E + 06 cells/mL and yielded viral vector titers up to 8.6E + 06 TU/mL. Despite the huge importance of HEK293-F cells as host in viral vaccines, viral vectors, and exosomes production (Le Ru et al. 2010; Petiot et al. 2015; Venereo-Sanchez et al. 2019; Zhao et al. 2020), surprisingly little effort went into the establishment of HCD cultivations for this cell line. Recently, in one of the first studies concerning this topic, the cultivation of HEK293-F cells in a steady state at 20.0E + 06 or 80.0E + 06 cells/mL was shown (Schwarz et al. 2020). These very high cell concentrations were achieved by optimization of the aeration conditions in a small scale bioreactor system (250 mL working volume) and ultimately revealed the potential of HEK293-F cells for HCD perfusion cultivations. Our results give further evidence for the suitability of HCD HEK293-F cultivations, especially for the production of viral vectors.

### The VHU allowed continuous harvesting of MLV vectors

Two different cell retention membranes were investigated for their potential to allow a continuous harvesting of MLV vectors; a commonly used HFM and a novel VHU. In previous studies, the used HFM (pore size of 0.2 µm) retained most virus particles with a size of about 100 nm (Genzel et al. 2014; Hein et al. 2021a; Nikolay et al. 2020). The VHU (pore size about 10 µm), however, was shown to allow continuous harvesting of influenza A virus particles (size about 100 nm) (Hein et al. 2021a). The results presented here are in line with these results. More specifically, the used HFM retained most of the produced MLV vectors (about 100 nm), but no viral vector retention was observed when the VHU was used. Continuous viral vector harvesting does not only allow the establishment of continuous HCD processes, but also the implementation of strategies, where virus particles are directly forwarded to downstream operation units. Such a process integration can offer several advantages including a reduction in product degradation due to shorter residence times, and reduced production costs (Granicher et al. 2021; Konstantinov and Cooney 2015).

The possibility to continuously harvest virus particles was already shown for other cell retention devices, such as the acoustic settler and the inclined settler (Ansorge et al. 2011; Coronel et al. 2020; Granicher et al. 2020). Many parameters must be considered to determine which cell retention system is best suited for a given application and a detailed comparison can be found elsewhere (Chotteau 2015). However, both the acoustic settler and the inclined settler have drawbacks such as the need to implement more complex cooling or pumping regimes. Also, the scale-up of processes is more demanding compared to the establishment of an ATF system (Chotteau 2015; Coronel et al. 2020; Granicher et al. 2020). Therefore, the ATF system might be more suitable for industrial processes, especially when the VHU can enable a continuous viral vector harvesting. However, it should not be omitted that use of an ATF device can cause higher shear stress, which could result in slower cell growth. This was also observed in our experiments, were the doubling time of the cells increased after the perfusion phase was initiated. Furthermore, very recently, the option for continuous virus harvesting was also shown for another membrane-based cell retention system. Specifically, using the tangential flow depth filtration (TFDF) system from Repligen Corp, a continuous virus harvesting regime was established for lentiviral vector production (Tran and Kamen 2022).

One concern regarding the use of the VHU microporous membrane in our recent experiments was the observed leakage of cells, as typically full cell retention is expected in perfusion and was also seen in previous studies with the VHU (Hein et al. 2021a). As described above due to a change in membrane manufacturer during the COVID pandemic, the VHU membranes used in this study did not possess the correct membrane parameters with respect to pore size. This issue has now been solved and future membranes will have pore sizes of 10 µm as validated by mercury intrusion porosimetry (data not shown). Since in this study a cell bleed was part of the original plan, we do not consider it necessary to repeat our experiments with the newer membranes which meet the 10 µm pore size requirement. More important for this work is the ability to achieve an increased viral yield as a result of direct viral vector harvesting.

### Continuous HCD cultivation increased STY

In the present study, the STY of the perfusion cultivation was 9-fold and for the continuous HCD cultivation 18-fold higher than the average of the two conducted batch cultivations (based on viral vector titers determined in NIH/3T3 cells). While this improvement is already impressive, it should be considered that this is a very conservative estimation. More specifically, the turnover times (STR set-up and cleaning time) were not considered for the calculation of the STY. Thus, the effective STY (also considering turnover times) is potentially much lower. Further, due to the shorter process times of batch cultivations, the cumulated turnover times are usually higher than for perfusion cultivations (Bausch et al. 2019) resulting in a more pronounced decrease in the STY. Therefore, the difference in the effective STY of batch cultivations compared to the intensified cultivations is likely even higher than suggested here.

When the HFM was used, MLV vectors were retained in the bioreactor and the process needed to be terminated to harvest the MLV vectors at maximum titer. In this context, especially the viral vector stability has to be considered. Keeping the viral vectors in the STR, which probably contains a relative high concentration of cellular proteases, could result in viral vector degradation. This was previously described for other viruses (Genzel et al. 2010; Hein et al. 2021b; Petiot et al. 2011) and could also be observed in the presented study for batch cultivations, where the viral vector titers strongly decreased after the maximum titer was reached. In contrast, the VHU allowed the implementation of a continuous HCD production process with a steady state, where high numbers of MLV vectors could be harvested continuously and immediately cooled. The advantage of this could also be observed in the number of collected viral vectors, which was higher for the VHU and kept increasing when it already stagnated for the HFM. Likewise, we observed a 2-fold increase in the STY compared to the perfusion cultivation with the HFM. Furthermore, the STY of any continuous process increases the longer the steady state is maintained (Bausch et al. 2019). In this proof of concept study, we only achieved a steady state for 5 days. However, other groups were able to maintain a steady state for HEK293-F cells in HCD cultivations for up to 66 days (Schwarz et al. 2020). It was shown before, that the here used VPC-MSCV-EGFP cell line stably expresses MLV vectors for up to three months, when a selective pressure was applied (van Heuvel et al. 2021). Expression without application of any selective pressure was not investigated, so far. However, in case the steady state could be maintained for 66 days, the STY would have been approximately 5-fold and 49-fold higher compared to the perfusion cultivation and the conventional batch cultivations, respectively.

### mRNA levels did not correlate with vector titers

For all batch, perfusion, and continuous cultivations with DYN, the mRNA levels and the viral vector titers were determined. As expected, viral vector titers determined using either NIH/3T3 or COS-7mCAT cells were always higher for cultivations with higher maximum VCC. This was most obvious when shake flask batch and STR perfusion cultivations were compared. Here, titers in perfusion cultivations were up to one order of magnitude higher. In contrast, no pronounced differences in the mRNA levels of batch and perfusion or continuous cultivations were observed. It should be noted, that a variety of additional measurements have been conducted to exclude that these results were only caused by technical difficulties, e.g. matrix effects or differences in sample preparation. One hypothesis, for this surprising result, could be that not all produced mRNA molecules were efficiently packaged into MLV vectors. This is supported by the observation that the mRNA levels were always 1–3 orders of magnitude higher than the viral vector titers. This overestimation of the particle count determined by qPCR measurements was already reported earlier for retrovirus quantification (Geraerts et al. 2006). Therefore, most of the measured mRNA particles in the supernatant might be unpackaged and exposed to released cellular nucleases. Here, it can be speculated that higher cell concentrations also resulted in higher amounts of released nucleases. Therefore, in a HCD cultivation, the higher mRNA production rate might be accompanied with a higher mRNA degradation rate, which could explain why the mRNA levels were overall comparable to the batch cultivations. Moreover, the selective packaging of cellular RNAs by MLV particles was described before (Eckwahl et al. 2016; Onafuwa-Nuga et al. 2005; Rulli et al. 2007). Consequentially, the mRNA quantification was not a reliable tool to compare viral vector titers of material produced with different process regimes. However, it should be considered to further investigate the packaging of mRNA in MLV vectors. More specifically, a very common problem for viral vector production is the formation of empty particles not containing the gene of interest (Gagnon et al. 2021; Gao et al. 2014). Therefore, the intensified investigation of MLV vector purification and potentially the separation of empty and full MLV vectors could be an interesting prospect of future research.

### MLV and LV production for gene therapy

Retrovirus-derived viral vectors, like MLV or LV vectors, have tremendous potential to treat patients suffering from life-threatening diseases. One of the most remarkable applications is the use of LV vectors for modification of a patient’s T cells to express chimeric antigen receptors, creating the CAR-T system (Elsner and Bohne 2017; Salter et al. 2018). The biggest bottleneck of this technology is the high demand of clinical-grade viral vectors. Specifically, the required doses are usually 1E + 11–1E + 12 TU/patient (Ansorge et al. 2010; Park et al. 2018), while the average titers in the cell-culture based production are in the order of 1E + 6 TU/mL (Ansorge et al. 2009; Sanber et al. 2015; Tomas et al. 2018). Considering the required doses and the expected titer, at least one 100 L-batch cultivation would be required per patient. Until today the most common production system for LV vectors is the transient transfection of adherent HEK cells with the required plasmids. However, several research groups have already reported the generation of stable producer cell lines for LV vector production (Farson et al. 2001; Sanber et al. 2015; Tomas et al. 2018). Using such a stable producer cell line would allow the application of the process intensification strategies outlined in the presented work. In this scenario, one 100 L-STR operated as a HCD continuous cultivation could generate enough material for the treatment of one patient every 6 h. Overall, our data contribute to the development of more efficient processes that are urgently needed to overcome the bottlenecks currently observed for the production of viral vectors.

## Supplementary Information

Below is the link to the electronic supplementary material.

Supplementary file1 (PDF 248 KB)

## Data Availability

The datasets generated and analyzed during the current study are available from the corresponding author on reasonable request.
