# Supplementary material for: Production of retroviral vectors in continuous high cell density culture
Source: Appl Microbiol Biotechnol. 2023 Aug 5;107(19):5947–61. doi: 10.1007/s00253-023-12689-9 (PMC10485120; doi:10.1007/s00253-023-12689-9)
Supplement: Supplementary file 1 — Supplementary file1 (PDF 248 KB) [file 253_2023_12689_MOESM1_ESM.pdf]

Journal: "Applied Microbiology and Biotechnology"

Manuscript Title: "Production of retroviral vectors in continuous high cell density culture"

Marc D. Hein[1], Daniel Kazenmaier[2,3], Yasemin van Heuvel[4,5], Tanya Dogra[2], Maurizio Cattaneo[6], Sascha Y. Kupke[2], Jörn Stitz[4], Yvonne Genzel[2,*], Udo Reichl[1,2]

[1]Otto-von-Guericke-University Magdeburg, Chair of Bioprocess Engineering, Magdeburg, Germany

[2]Max Planck Institute for Dynamics of Complex Technical Systems, Bioprocess Engineering, Magdeburg, Germany

[3]University of Applied Sciences Mannheim, Faculty of Biotechnology, Mannheim, Germany

[4]University of Applied Sciences Cologne, Faculty of Applied Natural Sciences, Leverkusen, Germany

[5]Institute of Technical Chemistry, Leibniz University Hannover, Hannover, Germany

[6]Artemis Biosystems, Cambridge, Massachusetts, USA

*** Correspondence:**
Yvonne Genzel
E-mail: genzel@mpi-magdeburg.mpg.de
Phone: +49 391 6110-257
Fax: +49 391 6110-565

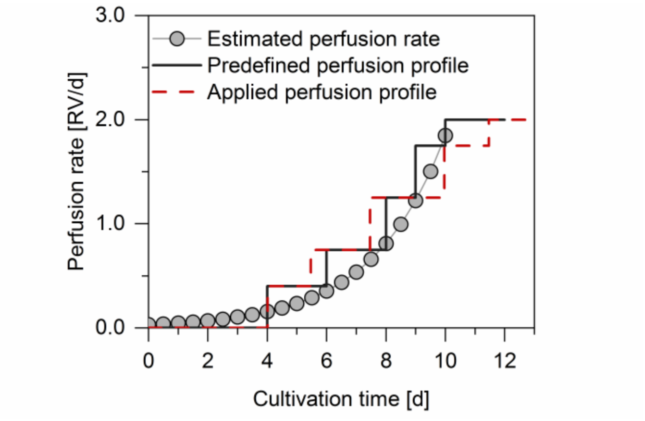

Fig. S1 Estimated perfusion rate, predefined, and applied perfusion profile for cultivation of VPC-MSCV cells in a 1 L stirred tank bioreactor coupled to an alternating tangential flow filtration system. For the calculation of the required perfusion rate, the maximum specific growth rate (0.0354 1/h) and the glucose uptake rate (3.62E–10 mmol/cell/h) of a batch previous cultivation (Fig. 2) was used. Considering the metabolite uptake rates, the glucose (40 mmol/L), and glutamine (8 mmol/L) concentration in the Dynamis medium, a cell specific perfusion rate (CSPR) of 60 pL/cell/d was calculated. Based on the expected cell growth and the CSPR the required perfusion rate was estimated. A predefined stepwise perfusion profile was chosen accordingly. During the cultivation, deviations to the expected cell growth were observed and the applied perfusion profile was adjusted accordingly.
